# Predictors of Spontaneous Pharyngocutaneous Fistula Closure After Total Laryngectomy

**DOI:** 10.7759/cureus.114539

**Published:** 2026-08-14

**Authors:** Ernesto Sánchez-LLanos, Rafael Fernández-Liesa, Eugenio A Vicente-González

**Affiliations:** 1 Otolaryngology-Head and Neck Surgery, Hospital Universitario Miguel Servet, Zaragoza, ESP; 2 Otolaryngology-Head and Neck Surgery, Universidad de Zaragoza, Zaragoza, ESP

**Keywords:** conservative management, pharyngocutaneous fistula, radiotherapy, spontaneous closure, total laryngectomy

## Abstract

Objective: This study aimed to evaluate the rate of spontaneous pharyngocutaneous fistula (PCF) closure after total laryngectomy and to identify clinical, inflammatory, radiotherapy-related, and perioperative factors associated with successful conservative management.

Materials and methods: A retrospective observational study was conducted, including patients who underwent total laryngectomy between 2010 and 2025. Clinical, surgical, and oncological variables were collected from medical records. Variables analyzed included demographic characteristics, tumor-related factors, previous treatments, radiotherapy parameters, perioperative laboratory values, surgical details, and postoperative complications. Among patients who developed PCF, fistula characteristics, spontaneous closure, and time to closure were assessed. Univariate and multivariable analyses were performed to identify predictors of spontaneous fistula closure.

Results: A total of 194 patients were included. PCF developed in 101 patients (52.3%), of whom 66 (65.3%) achieved spontaneous closure with conservative management. Fistula size was the strongest predictor of healing: 84.2% of punctate fistulas closed spontaneously compared with only 8% of large fistulas (p < 0.001; OR 61.3, 95% CI 12.7-295). Hemoglobin levels below 13 g/dL (p = 0.029; OR 2.64, 95% CI 1.09-6.35) and a platelet-to-lymphocyte ratio (PLR) >150 (p = 0.039; OR 2.61, 95% CI 1.03-6.60) were associated with reduced rates of spontaneous closure. Additional factors associated with persistent fistula included radiotherapy within the previous year, hypofractionated radiotherapy, higher acute radiation toxicity, intraoperative use of a Montgomery® salivary bypass tube (MSBT; Boston Medical Products, Westborough, MA, USA), and postoperative complications (infection or bleeding). Kaplan-Meier analysis demonstrated a significantly delayed time to spontaneous closure in patients with a PLR >150 (median 31 vs. 20 days; p = 0.026) and in those with a history of prior radiotherapy (median 25 vs. 17.5 days; p = 0.038). Multivariable analysis identified hemoglobin <13 g/dL, fistula size, intraoperative salivary bypass tube placement, and postoperative complications as independent predictors of failure of spontaneous closure. The predictive model demonstrated good discrimination (area under the receiver operating characteristic curve (AUC) 0.928) and 80% overall accuracy.

Conclusion: Approximately two-thirds of PCFs after total laryngectomy closed spontaneously under conservative treatment. Fistula size was the most important determinant of healing, while anemia, increased radiation-related toxicity, salivary bypass tube use, and postoperative complications independently predicted persistent fistula. Early identification of these factors may facilitate risk stratification and guide individualized management strategies, helping to optimize the timing of reconstructive intervention.

## Introduction

Pharyngocutaneous fistula (PCF) remains one of the most common and clinically significant complications following ablative head and neck surgery, particularly after total laryngectomy. Its occurrence has a substantial impact on postoperative recovery, leading to prolonged hospitalization [1,2], delayed initiation of oral feeding [3], postponement of adjuvant therapies [1,2], and a significant increase in morbidity, mortality [4], and healthcare costs [5].

The incidence of PCF remains high, with reported rates ranging from 5% to 65% depending on the study population and associated risk factors [6]. Among the main predisposing factors identified are previous radiotherapy [5], malnutrition [5,7], anemia [6,7], hypoalbuminemia [5,7], salvage surgery [5,8], local infection [8], and excessive tension on the pharyngeal closure [9].

Once a fistula develops, considerable variability exists regarding its clinical course and the need for additional surgical intervention. Spontaneous closure of PCF is of particular clinical interest, as it may obviate the need for complex reconstructive procedures and reduce the overall healthcare burden. However, the actual likelihood of spontaneous resolution remains a matter of debate. While some patients achieve satisfactory closure through conservative management, including local wound care, nutritional support, and infection control [10,11], others develop persistent fistulas requiring surgical reconstruction with regional or free flaps.

Identifying factors associated with spontaneous closure may be crucial for optimizing therapeutic decision-making and establishing individualized management strategies. Variables such as fistula size, nutritional status, previous radiotherapy, timing of postoperative onset, and the presence of necrotic tissue may significantly influence the probability of spontaneous healing; however, the currently available evidence remains limited.

Therefore, the aim of the present retrospective observational study is to analyze the main clinical and therapeutic factors associated with the likelihood of spontaneous closure of PCF following its development. A better understanding of these factors may contribute to more precise management strategies, reduce unnecessary interventions, and improve both functional and oncological outcomes in patients undergoing total laryngectomy.

## Materials and methods

Study design

A retrospective, observational, non-randomized, and non-blinded study was conducted, including all patients who underwent total laryngectomy at Hospital Universitario Miguel Servet, Zaragoza, Spain, between January 2010 and December 2025.

Study population

A total of 200 patients were initially identified. Six patients were excluded: two died during hospitalization due to postoperative complications, and four were excluded because of incomplete clinical data. The final study cohort consisted of 194 patients. All patients underwent comparable preoperative and postoperative management according to standardized institutional perioperative care protocols.

Objectives

The primary objective was to characterize patients undergoing total laryngectomy and to identify factors associated with the development of PCF. Among patients who developed PCF, we subsequently analyzed potential factors influencing spontaneous fistula closure.

The secondary objective was to identify potentially modifiable factors that could enhance the likelihood of spontaneous PCF closure.

Variables collected

Relevant clinical and surgical variables were retrospectively collected from medical records. The variables analyzed included age, sex, primary tumour location, tumour stage, extent of surgery, previous treatment (radiotherapy and/or chemotherapy), type and technique of pharyngeal closure, suture material, presence of previous tracheostomy, surgical margin status, preoperative haemoglobin and albumin levels, and postoperative complications.

Additionally, specific PCF-related variables were recorded, including fistula size, occurrence of spontaneous closure, and time to spontaneous closure.

PCF closure was clinically defined as the complete absence of salivary or oral intake leakage upon oral feeding, accompanied by complete mucosal healing confirmed by physical examination. Conservative management was maintained for a maximum duration of 60 days from PCF onset; however, an extension of up to an additional 30 days (maximum 90 days total) was allowed in cases showing favorable clinical evolution with progressive reduction of fistula output. Conservative treatment was considered unsuccessful if active leakage persisted beyond these timeframes. Surgical repair was indicated upon failure of conservative management, in high-output fistulas with extensive tissue loss, or in the presence of severe complications such as vessel exposure or local tissue necrosis.

Statistical analysis

Statistical analyses were performed using jamovi software (version 2.6.44.0; The jamovi project, Sydney, Australia). Descriptive analyses were conducted first. Continuous variables were expressed as mean ± standard deviation (SD) or median and interquartile range (IQR), depending on data distribution, which was assessed using the Shapiro-Wilk normality test. Categorical variables were summarized as absolute frequencies and percentages.

For bivariate analyses, the chi-square (χ²) test or Fisher's exact test was used for categorical variables, while Student's t-test or the Mann-Whitney U test was applied to continuous variables as appropriate. Statistical significance was defined as a two-sided p-value < 0.05.

Time to spontaneous fistula closure was analyzed using Kaplan-Meier survival curves. Closure of the PCF was considered the event of interest, and time was calculated from fistula diagnosis to documented spontaneous closure. Differences between groups were assessed using the log-rank test. Median time to closure and corresponding 95% confidence intervals (95% CI) were estimated from the Kaplan-Meier curves. A two-sided p-value < 0.05 was considered statistically significant.

A multivariate analysis was also performed including all variables that reached statistical significance in the bivariate analysis. Only patients with complete dosimetric and toxicity data were included in the multivariate analysis. No major clinical differences were identified between included and excluded patients; however, selection bias cannot be completely excluded.

Ethical considerations

This study was reviewed and approved by the Ethics Committee for Research of the Community of Aragón (Comité de Ética de la Investigación de la Comunidad de Aragón (CEICA)), with the reference number C.P.-C.I. PI20/213.

## Results

A total of 194 patients were included in the study (18 women and 176 men), with a mean age of 67.3 years (range 42-91 years; SD 10.9). The incidence of PCF was 101 (52.3%). Among these 101 patients, spontaneous fistula closure occurred in 66 cases (65.3%).

The mean length of hospital stay was 39 days among patients who achieved spontaneous closure compared with 66 days among those who did not, representing a statistically significant difference (Welch's t-test = 3.96, df = 38.7, p < 0.001).

Fistula size and spontaneous closure

**Figure 1:**
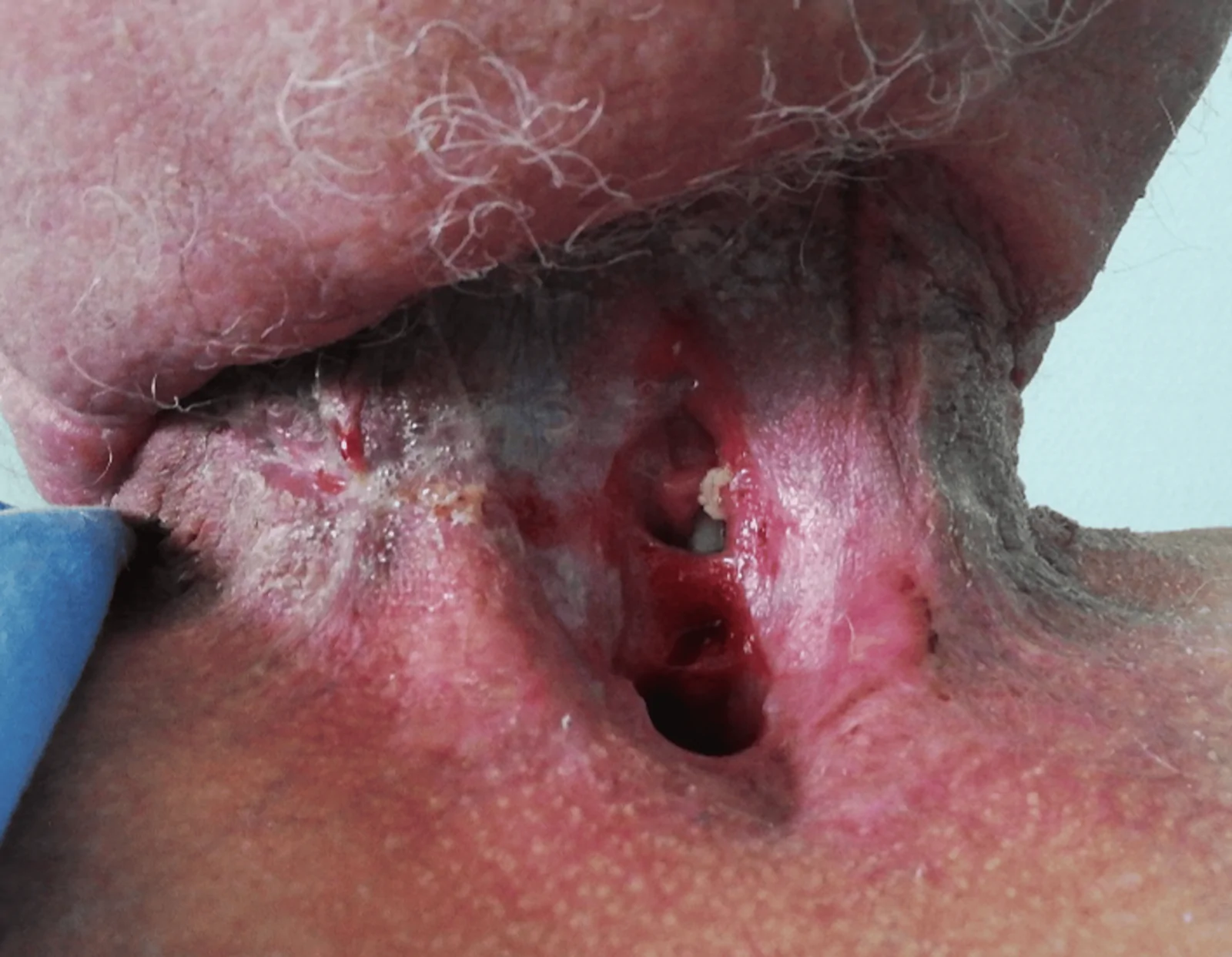
A large fistula clearly visible on inspection, exposing the pharyngeal cavity.

PCFs were classified as either punctate or large. A punctate fistula was defined as a clinically imperceptible or nearly imperceptible defect in which salivary leakage became evident only during swallowing (Video 1). In contrast, a large fistula was visible on direct inspection, allowing visualization of the pharyngeal structures through the defect (Figure 1).

**Video 1:** A punctate fistula that is not visible on routine inspection but demonstrates salivary leakage after milk ingestion.

Of the 101 PCFs, 76 (75.2%) were classified as punctate (Video 1), and 64 of these (84.2%) achieved spontaneous closure. Twenty-five fistulas (24.8%) were classified as large (Figure 1), with spontaneous closure occurring in only two cases (8%). The association between fistula size and spontaneous closure was highly significant (Pearson's χ² test = 48.2, df = 1, p < 0.001; OR 61.3, 95% CI 12.7-295)

Preoperative factors

Among preoperative variables, hemoglobin levels below 13 g/dL were significantly associated with a reduced likelihood of spontaneous closure (Pearson's χ² test = 4.78, df = 1, p = 0.029; OR 2.64, 95% CI 1.09-6.35).

**Figure 2:**
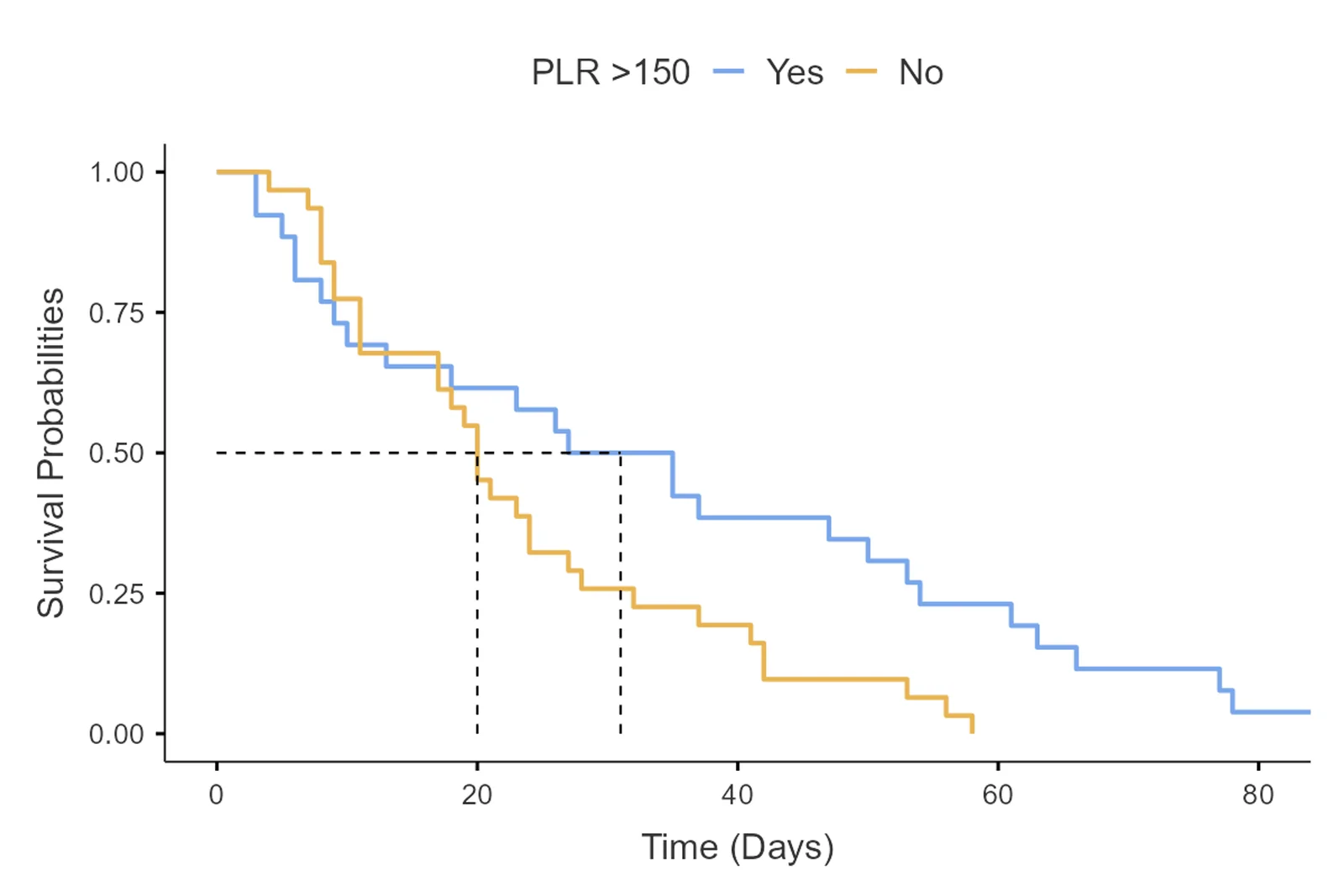
Kaplan–Meier curve showing time to spontaneous pharyngocutaneous fistula closure according to platelet-to-lymphocyte ratio (PLR). Patients with a PLR >150 demonstrated significantly delayed spontaneous fistula closure compared with those with a PLR ≤150. Survival curves differed significantly between groups (log-rank χ² = 4.97, df = 1, p = 0.026), indicating that increased systemic inflammation was associated with delayed wound healing and prolonged time to spontaneous fistula closure.

A platelet-to-lymphocyte ratio (PLR) >150, reflecting increased systemic inflammation, was also associated with a lower probability of spontaneous closure (Pearson's χ² test = 4.25, df = 1, p = 0.039; OR 2.61, 95% CI 1.03-6.60). Kaplan-Meier analysis demonstrated that patients with a PLR >150 experienced significantly delayed spontaneous closure of PCFs compared with those with a PLR ≤150. The median time to closure was 31 days (95% CI: 13-54) in patients with PLR >150, compared with 20 days (95% CI: 17-28) in those with PLR ≤150. Survival curves differed significantly between groups (log-rank χ² = 4.97, df = 1, p = 0.026), indicating that increased systemic inflammation was associated with delayed wound healing and prolonged time to spontaneous fistula closure (Figure 2).

Radiotherapy-related factors

Several radiotherapy-related variables were associated with reduced rates of spontaneous fistula closure. Although prior radiotherapy itself did not reach statistical significance, it approached significance (p = 0.084).

Patients who had received radiotherapy within the previous year were more likely to experience persistent fistulas without spontaneous closure (Pearson's χ² test = 5.07, df = 1, p = 0.024; OR 3.35, 95% CI 1.15-9.73).

Hypofractionated radiotherapy (>2 Gy per fraction) was associated with a significantly lower likelihood of spontaneous closure compared with conventional fractionation schedules (Pearson's χ² test = 9.89, df = 1, p = 0.002; OR 6.84, 95% CI 1.96-23.9).

Radiotherapy planning technique also influenced outcomes. Intensity-modulated radiotherapy (IMRT) was associated with significantly lower spontaneous closure rates than three-dimensional conformal radiotherapy (Pearson's χ² test = 14.8, df = 1, p < 0.01; OR 11.9, 95% CI 3.06-46.0).

Acute radiotherapy-related toxicity was graded according to the Radiation Therapy Oncology Group/European Organisation for Research and Treatment of Cancer (RTOG/EORTC) toxicity criteria [12,13]. In our cohort, patients presenting toxicity scores >1 had significantly lower rates of spontaneous closure (Pearson's χ² test = 4.39, df = 1, p = 0.043; OR 3.86, 95% CI 1.05-14.2).

**Figure 3:**
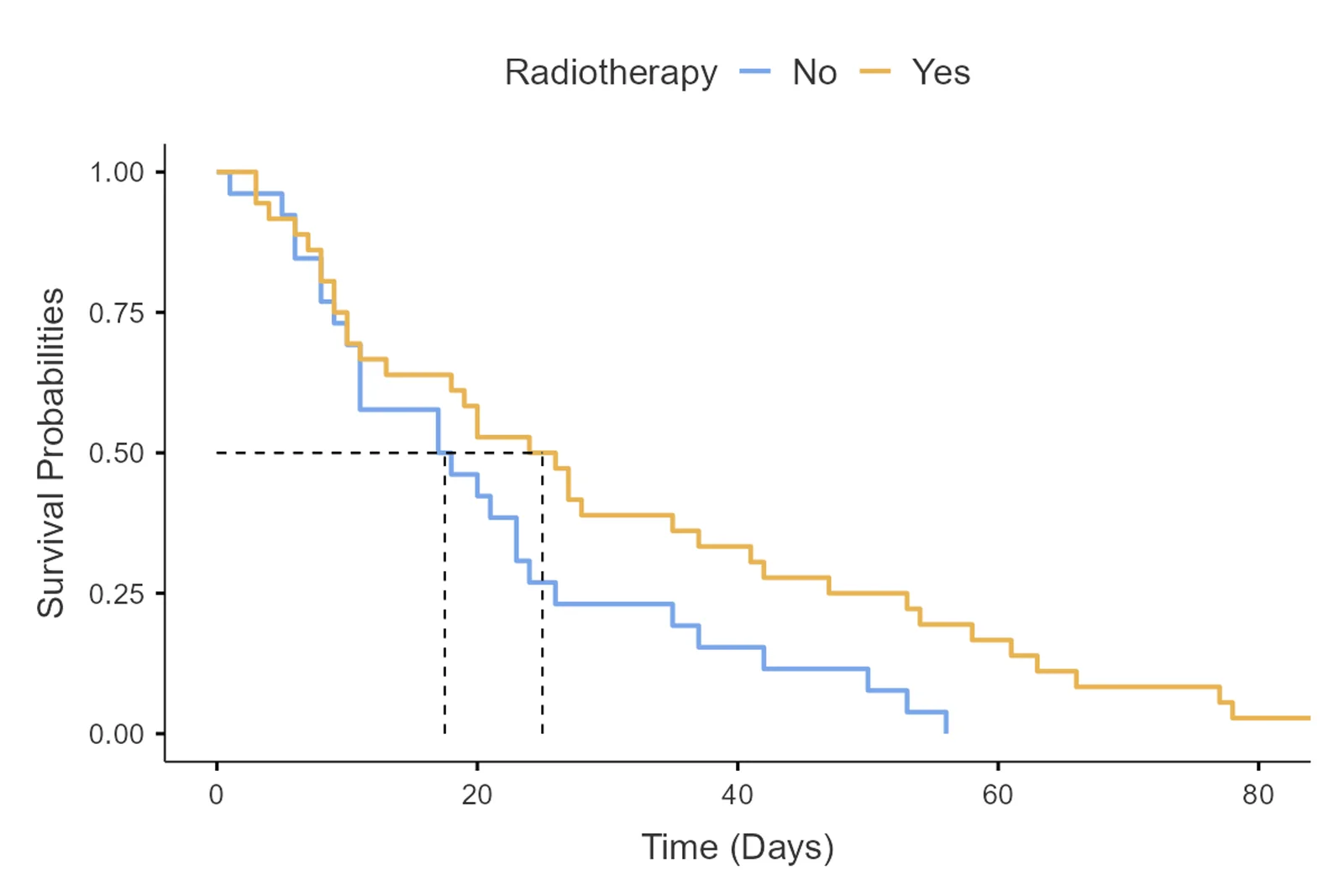
Kaplan–Meier curve showing time to spontaneous pharyngocutaneous fistula closure according to previous radiotherapy status. Patients with a history of radiotherapy experienced significantly delayed spontaneous fistula closure compared with non-irradiated patients. Survival curves differed significantly between groups (log-rank χ² = 4.30, df = 1, p = 0.038), indicating that previous radiotherapy was associated with a prolonged healing process and delayed spontaneous fistula closure.

Kaplan-Meier analysis demonstrated that patients with a history of radiotherapy experienced significantly delayed spontaneous closure of PCFs compared with non-irradiated patients. The median time to closure was 25 days (95% CI: 13-41) in irradiated patients, compared with 17.5 days (95% CI: 11-26) in non-irradiated patients. Survival curves differed significantly between groups (log-rank χ² = 4.30, df = 1, p = 0.038), indicating that previous radiotherapy was associated with a prolonged healing process and delayed spontaneous fistula closure (Figure 3).

Surgical factors

The intraoperative use of a Montgomery® salivary bypass tube (MSBT; Boston Medical Products, Westborough, MA, USA) was associated with a lower likelihood of spontaneous closure (Pearson's χ² test = 9.31, df = 1, p = 0.020; OR 4.20, 95% CI 1.62-10.9).

Postoperative complications

Postoperative wound infection or bleeding significantly reduced the probability of spontaneous closure (Pearson's χ² test = 6.05, df = 1, p = 0.014; OR 3.33, 95% CI 1.25-8.91).

Multivariable analysis

A multivariable binary logistic regression model was developed to identify independent predictors of spontaneous PCF closure (Table 1). The model showed a deviance of 39.7, an Akaike Information Criterion (AIC) of 48.2, and a McFadden's R² of 0.537, indicating satisfactory explanatory performance for this type of multivariable model.

**Table 1:** Multivariate logistic regression analysis of independent predictors for pharyngocutaneous fistula closure Statistical significance was defined as p < 0.05. Significant p-values are indicated with an asterisk (*). Independent predictors of failure of spontaneous closure included postoperative hemoglobin <13 g/dL (p = 0.012), fistula size (p < 0.001), intraoperative use of Montgomery® salivary bypass tube (MSBT; p = 0.029), and the occurrence of unexpected postoperative complications (infection or bleeding) (p = 0.012). AIC: Akaike information criterion; SE: standard error

Model	Deviation	AIC	McFadden's R²				
1	35.2	45.2	0.365				
Note: Models estimated using a sample size of N=74							
Predictor	Estimate	SE	Z	p	OR	95% CI OR	
Hemoglobin <13 (No-Yes)	1.24	0.779	1.59	0.012*	3.44	1.19	15.85
Fistula size (Punctate-Large)	4.05	0.907	4.46	<0.001*	57.29	9.67	339.28
MSBT (No-Yes)	1.90	0.868	2.19	0.029*	7.16	1.55	33.14
Complication (No-Yes)	1.97	0.782	2.52	0.012*	6.68	1.22	36.57

Independent predictors of failure of spontaneous closure included postoperative hemoglobin <13 g/dL (p = 0.012), fistula diameter size (p < 0.001), intraoperative use of MSBT (p = 0.012), and the occurrence of unexpected postoperative complications (infection or bleeding) (p = 0.029).

**Figure 4:**
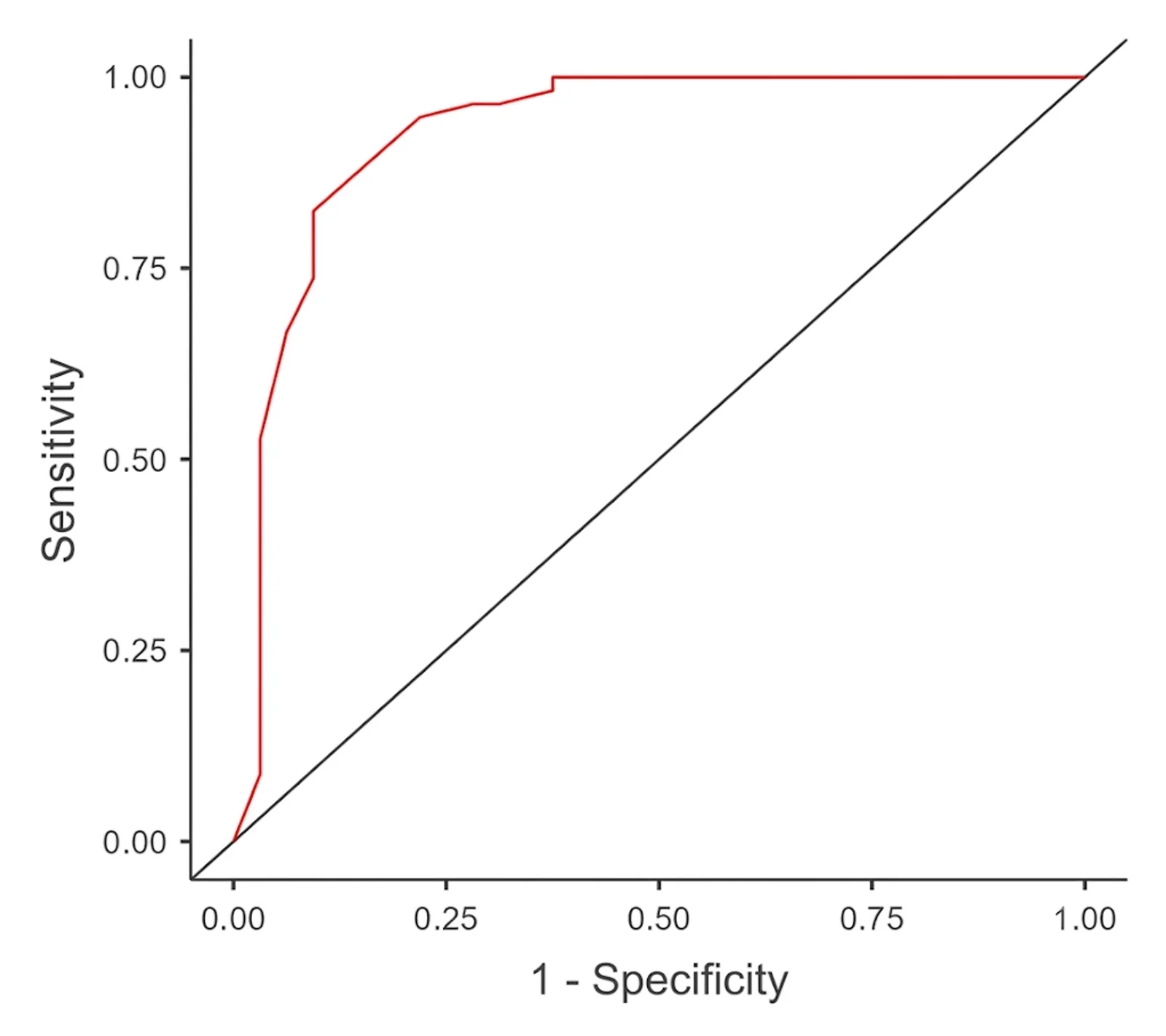
Receiver operating characteristic (ROC) curve for prediction of pharyngocutaneous fistula. The area under the ROC curve (AUC) was 0.928, indicating good discriminative performance.

Using a probability threshold of 0.5, the model achieved an overall accuracy of 88%, with sensitivity of 0.947 and specificity of 0.781. The area under the receiver operating characteristic curve (AUC) was 0.928, indicating good discriminative performance (Figure 4).

## Discussion

PCF remains one of the most clinically significant complications following total laryngectomy, particularly in the setting of salvage surgery. In our series, the overall incidence of PCF was 52.3%, a relatively high rate but consistent with previous reports in patients who had undergone prior radiotherapy and complex head and neck surgery. However, one of the most relevant findings of the present study was that approximately two-thirds of PCFs achieved spontaneous closure with conservative management, in agreement with previously published studies [2, 14]. This finding highlights that not all fistulas exhibit the same clinical behavior and that surgical reconstruction is not invariably required [5].

Among all variables analyzed, fistula size demonstrated the strongest association with spontaneous closure. Punctate PCFs exhibited substantially higher spontaneous healing rates than large fistulas. This finding is clinically plausible, as smaller fistulas generally preserve greater vascular and tissue integrity, allowing progressive healing through conservative measures. In contrast, large fistulas involve substantial tissue loss and continuous exposure to saliva and bacterial contamination, impairing granulation tissue formation and promoting chronicity of the defect. These results support the need for early assessment of fistula size to guide therapeutic decision-making and avoid unnecessary delays in reconstructive intervention.

Another important finding was the influence of hematological status and systemic inflammatory profile on spontaneous closure. Patients with hemoglobin levels below 13 g/dL showed a significantly lower probability of spontaneous fistula resolution. Anemia reduces tissue oxygen delivery and compromises wound-healing mechanisms, particularly in previously irradiated tissues with impaired vascularization [15]. Similarly, an elevated PLR was associated with poorer fistula outcomes. This marker has previously been linked to persistent systemic inflammation and dysregulated immune responses, factors that could interfere with tissue repair and promote persistence of the pharyngocutaneous communication [16]. To our knowledge, no previous studies have specifically demonstrated a direct association between hypoxia or systemic inflammation and reduced rates of spontaneous PCF closure. Another relevant finding was the influence of systemic inflammatory status on fistula healing dynamics. Patients with a PLR greater than 150 experienced a significantly longer time to spontaneous closure compared with those with lower PLR values. In addition to being associated with a lower overall likelihood of spontaneous closure in the bivariate analysis, an elevated PLR was also associated with delayed healing among patients who ultimately achieved fistula closure. Our findings suggest that systemic inflammation may influence not only the development of fistulas but also their subsequent healing trajectory and the time required for spontaneous closure. This observation highlights the potential value of inflammatory biomarkers for risk stratification and supports the importance of optimizing perioperative inflammatory and nutritional status in patients undergoing salvage laryngectomy.

Previous radiotherapy remains one of the most extensively debated factors regarding both the development and subsequent evolution of PCF [9]. In our cohort, a history of radiotherapy alone did not reach statistical significance, although a clear trend was observed. Nevertheless, several radiotherapy-related variables were significantly associated with reduced rates of spontaneous closure. An interval of less than one year between completion of radiotherapy and salvage surgery was associated with poorer outcomes, likely because microvascular injury, fibrosis, and tissue hypoxia are more pronounced during the early post-irradiation period [17]. Likewise, hypofractionated radiotherapy was associated with a significantly lower probability of spontaneous closure. This treatment modality delivers higher doses per fraction, increasing late toxicity in normal tissues and promoting permanent vascular damage.

One of the most striking findings was the association between IMRT and a lower probability of spontaneous closure. Although IMRT is currently considered the standard radiotherapy technique due to its ability to reduce toxicity to organs at risk [18], this association may be explained by the fact that IMRT is more frequently used in patients with advanced disease or extensive nodal involvement requiring broader target coverage [19]. Therefore, the observed relationship may reflect greater clinical complexity and more demanding dosimetric requirements rather than a direct detrimental effect of the technique itself. Nonetheless, this finding emphasizes the importance of evaluating not only the administration of radiotherapy but also its specific dosimetric and clinical characteristics.

Acute radiotherapy-related toxicity, assessed according to the RTOG/EORTC classification [12,13], was also significantly associated with persistent PCF. Patients with toxicity grades >1 had a significantly lower likelihood of spontaneous closure. This observation is particularly noteworthy because acute toxicity may act as an indirect marker of tissue radiosensitivity and severe mucosal injury [18]. The presence of severe mucositis [20], xerostomia, dysphagia, or radiation dermatitis may reflect diminished regenerative capacity of irradiated tissues [21], thereby impairing postoperative wound healing.

In addition to its well-established role as a risk factor for PCF formation, our findings suggest that previous radiotherapy may also influence the subsequent healing process once a fistula has developed. Irradiated patients experienced a significantly longer time to spontaneous closure, with a median healing time of 25 days compared with 17.5 days in non-irradiated patients.

To date, radiotherapy characteristics, dosimetric parameters, and treatment-related complications have been poorly investigated as determinants of both PCF development and subsequent spontaneous closure. While radiotherapy has been extensively investigated as a risk factor for PCF development, our results suggest that it may also affect the natural history of established fistulas by delaying spontaneous healing. This finding may have practical implications when counseling patients and determining the optimal timing for reconstructive intervention.

Regarding the intraoperative use of the MSBT, our results demonstrated a negative association with spontaneous closure. Although this device is commonly employed as a protective measure in high-risk patients, its use is likely influenced by the surgeon’s intraoperative assessment of increased reconstructive difficulty or poor tissue quality [22]. Consequently, MSBT may function more as a surrogate marker of surgical complexity than as a direct causal factor [23].

The occurrence of postoperative complications such as wound infection or bleeding was another factor that significantly reduced the probability of spontaneous closure. Both events promote local inflammation, bacterial contamination, and disruption of vascular supply within the surgical bed, thereby impairing wound healing [10].

Multivariable analysis identified four independent predictors associated with persistent PCF: hemoglobin levels below 13 g/dL, fistula size, intraoperative use of MSBT, and the occurrence of postoperative complications. The model demonstrated good predictive performance, with an AUC of 0.927 and an overall accuracy of 88%, suggesting potential clinical utility for identifying patients at increased risk of unfavorable fistula evolution.

This study has several limitations. First, its retrospective, single-center design is inherently susceptible to selection and information biases, and the generalizability of the findings to other institutional settings should be interpreted with caution. Importantly, given the observational nature of the study, the observed associations, particularly regarding IMRT, mucositis status (MSBT), and the PLR, reflect statistical correlations and do not necessarily imply direct causal relationships. Second, the multivariable analysis was restricted to 88 of the 101 patients who developed PCF, owing to missing dosimetric and toxicity data in the remaining cases. Although no major baseline differences were identified between included and excluded patients, residual selection bias cannot be entirely excluded. This sample size reduction is directly reflected in the wide CIs observed for several independent predictors, which limit the precision of the point estimates and call for cautious interpretation. Furthermore, the relatively limited sample size carries a potential risk of model overfitting, meaning that the predictive performance of our multivariable model should be interpreted with prudence. Third, fistula size was classified dichotomously based on clinical assessment by the operating or treating surgeon, without a standardized, objective measurement method such as fistula diameter in millimeters or a validated grading scale. This pragmatic approach, while consistent throughout the series, may introduce interobserver variability and limits direct comparability with other published cohorts. Future studies should consider adopting a validated, reproducible classification system to enable more rigorous cross-center comparisons. Finally, although key systemic markers were recorded, comprehensive preoperative and postoperative nutritional parameters (such as detailed serum protein panels or validated nutritional screening scores) were not systematically available, representing potential unmeasured confounding factors that could influence tissue healing and closure kinetics.

Despite these limitations, the present study provides relevant insights into factors associated with spontaneous PCF closure following total laryngectomy. The combination of clinical, inflammatory, and radiotherapy-related variables contributes to a better understanding of the biological behavior of these fistulas. However, any proposed predictive framework or model derived from these data requires prospective, external validation in independent cohorts before it can be reliably implemented in routine clinical practice. Future prospective multicenter studies will be necessary to validate these findings and establish individualized prevention and treatment strategies.

## Conclusions

Approximately two-thirds of PCFs following total laryngectomy achieved successful closure with conservative management, highlighting that surgical reconstruction is not required in all cases. Fistula size was the factor most strongly associated with healing, with punctate fistulas showing markedly higher closure rates than large defects.

Low hemoglobin levels, increased radiotherapy-related toxicity, intraoperative use of an MSBT, and postoperative complications were identified as independent predictors of persistent fistula. In addition, several radiotherapy-related characteristics, including a short interval between radiotherapy and surgery, hypofractionation, and IMRT-based treatment, were associated with poorer healing outcomes. These findings suggest that careful assessment of clinical, inflammatory, and radiotherapy-related factors may help stratify patients according to their likelihood of successful conservative management and facilitate earlier individualized treatment decisions. Prospective multicenter studies are warranted to validate these results and develop robust predictive models for clinical practice.
